# Analytical validation of a real-time hydrolysis probe PCR assay for quantifying *Plasmodium falciparum* parasites in experimentally infected human adults

**DOI:** 10.1186/s12936-021-03717-y

**Published:** 2021-04-10

**Authors:** Claire Y. T. Wang, Emma L. Ballard, Zuleima Pava, Louise Marquart, Jane Gaydon, Sean C. Murphy, David Whiley, Peter O’Rourke, James S. McCarthy

**Affiliations:** 1 Centre for Children’s Health Research, Children’s Health Queensland, Brisbane, Australia; 2 Child Health Research Centre, The University of Queensland, Brisbane, Australia; 3 QIMR Berghofer Medical Research Institute, Brisbane, Australia; 4 Departments of Laboratory Medicine and Microbiology, University of Washington, Seattle, WA USA; 5 Center for Emerging and Re-Emerging Infectious Diseases, University of Washington, Seattle, WA USA; 6 UQ Centre for Clinical Research, Faculty of Medicine, The University of Queensland, Brisbane, Australia; 7 School of Medicine, The University of Queensland, Brisbane, Australia

**Keywords:** *Plasmodium falciparum*, Hydrolysis probe, Quantitative PCR, Validation, Volunteer infection studies, MIQE

## Abstract

**Background:**

Volunteer infection studies have become a standard model for evaluating drug efficacy against *Plasmodium* infections. Molecular techniques such as qPCR are used in these studies due to their ability to provide robust and accurate estimates of parasitaemia at increased sensitivity compared to microscopy. The validity and reliability of assays need to be ensured when used to evaluate the efficacy of candidate drugs in clinical trials.

**Methods:**

A previously described 18S rRNA gene qPCR assay for quantifying *Plasmodium falciparum* in blood samples was evaluated. Assay performance characteristics including analytical sensitivity, reportable range, precision, accuracy and specificity were assessed using experimental data and data compiled from phase 1 volunteer infection studies conducted between 2013 and 2019. Guidelines for validation of laboratory-developed molecular assays were followed.

**Results:**

The reportable range was 1.50 to 6.50 log_10_ parasites/mL with a limit of detection of 2.045 log_10_ parasites/mL of whole blood based on a parasite diluted standard series over this range. The assay was highly reproducible with minimal intra-assay (SD = 0.456 quantification cycle (C_q_) units [0.137 log_10_ parasites/mL] over 21 replicates) and inter-assay (SD = 0.604 C_q_ units [0.182 log_10_ parasites/mL] over 786 qPCR runs) variability. Through an external quality assurance program, the QIMR assay was shown to generate accurate results (quantitative bias + 0.019 log_10_ parasites/mL against nominal values). Specificity was 100% after assessing 164 parasite-free human blood samples.

**Conclusions:**

The 18S rRNA gene qPCR assay is specific and highly reproducible and can provide reliable and accurate parasite quantification. The assay is considered fit for use in evaluating drug efficacy in malaria clinical trials.

**Supplementary Information:**

The online version contains supplementary material available at 10.1186/s12936-021-03717-y.

## Background

Enhanced global efforts aimed at malaria control and elimination have led to a significant decrease in the incidence of malaria over recent years [1]. However, the emergence of drug-resistant parasites [2–4] has the potential to undermine progress made to date and highlights the need for ongoing development of effective and affordable anti-malarial drugs and vaccines.

Volunteer infection studies (VIS), also known as controlled human malaria infection studies, afford a safe and reproducible model to assess anti-malarial drug efficacy at an early stage, and predict drug activity in natural infections [5–9]. They have become a standard model for early evaluation of the efficacy of candidate drugs or vaccines [7, 8, 10–12]. In VIS, participants are inoculated with sporozoites or blood stage parasites via mosquito bites or, injection [7, 8, 13]. A critical safety consideration in VIS is the parasite density in the peripheral blood of infected participants. Likewise, this metric is critical to determining the efficacy of vaccines and drugs acting against blood stage parasites. Traditionally, parasitaemia has been monitored via microscopy [14, 15]. However, microscopy has a limited sensitivity of ~ 20 parasites/µL blood in expert laboratories [16, 17] and can produce false-negative results in low-density or asymptomatic infections [18, 19].

Molecular techniques, such as quantitative PCR (qPCR) and reverse transcription qPCR (RT-qPCR), have become the preferred methodology for evaluating drug and vaccine efficacy in early phase clinical trials across many centres [7, 10, 20–22]. Notably, these methods provide robust and accurate quantification of parasitaemia with increased sensitivity compared to the traditional methods: for example, allowing the detection of parasites in blood 1 to 4 days earlier than microscopy [16, 22, 23]. These enhanced performance characteristics of qPCR also ensure the safety of VIS by reducing the risk of dangerous levels of parasitaemia and minimizing discomfort of participants [10, 24]. The increased sensitivity and dynamic range of qPCR also permits improved estimation of important parasite parameters, such as asexual parasite maturation and multiplication rate [25]; these measures enable more accurate statistical modelling to determine pharmacodynamics of new anti-malarial treatments and characterization of the minimum inhibitory concentration for dose optimization [23, 26, 27].

However, ongoing assay characterization and validation is important to ensure accurate results are reported and to support clinical trials under rigorous regulatory review. For this purpose, additional validation was performed to assess performance characteristics of a previously described 18S rRNA gene qPCR assay [28] for quantifying *Plasmodium falciparum* parasites in healthy volunteers enrolled in VIS. A different 18S rRNA qPCR assay was recently qualified for this purpose through the U.S. FDA [22]. Parameters assessed were according to published guidelines [29–32], namely analytical sensitivity, reportable range, precision, accuracy and specificity.

## Methods

### Plasmodium 18S rRNA gene qPCR assay

The 18S rRNA gene qPCR assay has been previously described [28]. In brief, 2 mL blood samples were collected into ethylenediaminetetraacetic acid (EDTA) tubes from malaria-naïve participants enrolled in VIS. Tubes were centrifuged for 5 min at 2500 rpm to pellet red blood cells (RBCs), packed RBCs (~ 250 µL) were removed and mixed thoroughly with 250 µL phosphate buffered saline (PBS) and subjected to DNA extraction using QIAamp DNA Blood Mini Kit (Qiagen, Australia), with a final eluate volume of 100 µL. Five microlitres of nucleic acid were added to the qPCR mix as per the previously published qPCR assay specific for the *P. falciparum* 18S rRNA gene [29, 30] using the Qiagen Rotor-Gene platform (Qiagen, Australia) and the Qiagen QuantiTect Probe mix (Qiagen, Australia.) Details of the primers and probe are presented in Additional file 1: Table S1.

### Controls and calibration verification

A reference standard for calibration was prepared from *P. falciparum* 3D7 ring-stage synchronized culture using methods previously described [28, 33]. The parasite density of the material was estimated by fluorescence-activated cell sorting to be 3.19 × 10^6^ parasites/mL (6.50 log_10_ parasites/mL) and this material was subjected to nucleic acid extraction. The extract was then serially diluted in uninfected human blood extracts to produce 5 tenfold dilutions ranging from 3.19 × 10^5^ to 31.9 parasites/mL (6.50 to 1.50 log_10_ parasites/mL). The six standards were analysed on a single qPCR run in duplicate to establish an external standard curve [34]. The linear regression model from an external standard curve (slope − 3.324) was then imported into all qPCR runs with fixing of the intercept to the highest standard concentration (6.50 log_10_ parasites/mL), a standardized calibration method. The remaining five standards were used during each subsequent qPCR and acted as positive controls, and for calibration verification by confirming adequate PCR efficiency (> 90%). A single sample of each concentration occurred with each qPCR run during clinical trials. Levey–Jennings charts were used to monitor quantification cycle (C_q_) fluctuations of positive samples [16, 29, 35]. Two negative controls were included in each qPCR run to monitor reagent contamination. An internal control targeting an equine herpesvirus (EHV) was used to monitor nucleic acid extraction efficiency and qPCR inhibition [28, 36]. Samples were spiked with a known concentration of the EHV prior to DNA extraction. C_q_ values obtained from the EHV PCR testing were considered acceptable if they fell within mean C_q_ ± 2 standard deviations (SD) of each extraction per qPCR run [37].

### Overview of 18S rRNA gene qPCR assay validation

The methodology used to validate the 18S rRNA gene qPCR assay followed the validation of laboratory-developed molecular assays for infectious diseases guidelines [29] and the minimum information for publication of quantitative real-time PCR (qRT-PCR) experiments [30]. Clinical and Laboratory Standards Institute guidelines were also used for guidance [31, 32]. The following characteristics of the 18S rRNA gene qPCR assay were assessed: analytical sensitivity, reportable range, precision including intra-assay and inter-assay variability, accuracy and specificity.

Nine standards ranging from 1.50 to 6.50 log_10_ parasites/mL were run on 3 consecutive days with seven technical replicates and a negative control. This dataset was used to determine the analytical sensitivity, reportable range and intra-assay variability. A linear regression model was used to estimate the relationship between log_10_ concentration of standard and C_q_ value. For this model technical replicates were averaged within days. The slope estimate was used to calculate qPCR efficiency. The intra-assay SD for each concentration was calculated using analysis of variance accounting for day using all technical replicate data. The overall SD is the pooled SD estimates across concentrations. Probit regression was used to estimate the limit of detection (LOD) at which 95% of the samples tested positive.

Inter-assay variability was assessed using the six standards data from 786 qPCR runs occurring during 30 VIS conducted between 2013 and 2019. The inter-assay SD for each concentration was calculated using analysis of variance accounting for study cohort. The overall SD is the pooled SD estimates across concentrations. The cohort SD for each concentration was calculated using analysis of variance accounting for study cohort and the interaction between study cohort and study. The overall cohort SD is the pooled SD estimates across concentrations. Data were checked for outliers and analysed using Stata version 14.2 (StataCorp, College Station, TX) or SPSS version 23.0 (IBM Corp., Armonk, NY). Data were summarized for each standard concentration by frequency and percentage detected, mean C_q_, SD, and relative variability of C_q_ measured as the percent coefficient of variation (%CV).

### Accuracy

To evaluate the accuracy of the 18S rRNA gene qPCR assay, the QIMR qPCR results were compared to the University of Washington reverse transcription quantitative PCR (RT-qPCR) via an external quality assurance (EQA) project [38]. This project was initiated in 2014 by the University of Washington to examine the qualitative and quantitative agreement between centres conducting VIS. The EQA panel comprised five level dilutions and negative controls preserved in 0.5 mL of frozen whole blood (n = 60) shipped to each laboratory on dry ice. The concentrations were originally de-identified, but later identified as high (5.48 log_10_ parasites/mL or 300,000 parasites/mL), mid (3.78 log_10_ parasites/mL or 6000 parasites/mL), low (2.78 log_10_ parasites/mL or 600 parasites/mL), very low (1.78 log_10_ parasites/mL or 60 parasites/mL), trace (0.78 log_10_ parasites/mL or 6 parasites/mL) and a negative control. The methods and results from five VIS centres participating in this program have been published [38]. The QIMR laboratory subsequently obtained this EQA panel and tested these with the QIMR 18S rRNA gene qPCR method. Data were reported as the parasite number detected and quantitative bias against nominal values and University of Washington values using Bland–Altman plots.

In addition, to estimate the impact of genetic diversity of the 18SrRNA gene on the assay performance, a search was conducted for single nucleotide polymorphisms (SNPs) in the area where primers and probe bind in 218 *P. falciparum* genomes in PlasmoDB (version 46, released the 6th Nov 2019) from 18 different countries in Asia, South-America and Africa.

### Specificity

Analytical specificity was previously determined to be 100% by comparing the sequence of the nucleic acid target to sequences available on publicly accessible databases using the NCBI BLAST search tool [37]. Here, an updated BLAST search was conducted to confirm specificity; briefly, primers and probe sequences were submitted using the nBLAST tool to check the assay’s specificity to *P. falciparum* and cross-matched against other *Plasmodium* species (including *Plasmodium ovale*, *Plasmodium malariae*, *Plasmodium knowlesi* and *Plasmodium vivax*) and human genomic DNA on 7th April 2020. Diagnostic specificity was evaluated by analysing qPCR assay on blood samples collected from malaria-naïve participants (n = 164) enrolled in 30 cohorts of induced blood stage malaria VIS during 2013 to 2019. Samples were collected from participants prior to being inoculated with blood-stage parasites.

Potential qPCR-interfering substances were considered, including endogenous substances such as haem, leukocyte DNA and plasma proteins in plasma [39, 40], and exogenous substances such as EDTA anticoagulant [39, 41]. The QIMR standard extraction protocol included the following procedures to avoid some known inhibitory substances: pelleting RBCs to remove plasma and buffy coat containing plasma proteins and leukocytes [28]. The nucleic acid extraction kit used was suitable for removing known inhibitory chemicals such as citrate, heparin and EDTA (QIAmp DNA Blood Mini Kit). An interference study was performed to examine the potential effect of haem (from haemoglobin) and EDTA from collection tubes on qPCR inhibition. Blood was collected in EDTA-coated tubes from healthy participants and subjected to centrifugation at 2500 rpm for 5 min. Plasma and buffy coat were removed and aliquots (n = 20) of 250 µL pelleted RBCs were mixed with 250 µL PBS and spiked with known concentration of *P. falciparum* culture and internal control EHV. A parallel extraction using 500 µL PBS (n = 20) with the same positive and EHV spikes was performed simultaneously. All extracts were analysed using 18S rRNA gene qPCR and EHV qPCR assays. C_q_ values from both assays were analysed using Student’s t-test to evaluate the effect of potential interference substances on the assay.

### Ethical approvals

Blood samples were collected during the course of VIS, which were all approved by the QIMR Human Research Ethics Committee. Details of the studies and ethical approval have been published ([42], Additional file 1: Table S2).

## Results

The overall performance characteristics of the 18S rRNA gene qPCR assay are summarized in Table 1. Details of each characteristic are given in the following section and in Additional file 1: Tables S1–S4.

**Table 1.** 18S rRNA gene qPCR performance characteristics

Analytical sensitivity	
Limit of detection (LOD)	2.045 log_10_ parasites/mL of whole blood
Lower limit of quantification (LLoQ)	1.50 log_10_ parasites/mL of whole blood
Upper limit of quantification (ULoQ)	6.50 log_10_ parasites/mL of whole blood
Reportable range	1.50 to 6.50 log_10_ parasites/mL of whole blood
Precision	
Intra-assay variability	Overall standard deviation (SD) = 0.456 C_q_ units (0.137 log_10_ parasites/mL)
Inter-assay variability	Overall standard deviation (SD) = 0.604 C_q_ units (0.182 log_10_ parasites/mL)
Accuracy	
EQA program	Bias: + 0.019 log_10_ parasites/mL against nominal parasitaemia values [38]
Specificity	
Analytical specificity	100%
Diagnostic specificity	100%

### Analytical sensitivity

The lowest concentration that could reliably be detected by the qPCR assay in ≥ 95% positive samples (LOD_95%_) as determined by Probit regression was 2.045 log_10_ parasites/mL (95% CI 1.875–2.591) or 111 parasites/mL (95% CI 75–390) of whole blood. The upper limit of quantification for the 18S rRNA gene qPCR assay (i.e., the highest standard of the reportable range [43] which was restricted to the highest concentration available in the laboratory) was 6.50 log_10_ parasites/mL. The lower limit of quantification (LLoQ; i.e., the lowest standard concentration that can be quantitatively determined with acceptable precision [43]) was 1.50 log_10_ parasites/mL of whole blood.

### Reportable range

A summary of C_q_ values for each standard dilution is presented in Additional file 1: Table S3. Two C_q_ values were identified as being outliers and changed to not-detected (ND); 2.20 log_10_ parasites/mL [C_q_ = 38.24] and 1.80 log_10_ parasites/mL [C_q_ = 42.98]) as they were visually aberrant being greater than the mean C_q_ of the standard concentration below and close to the maximum value of its range. Parasites were not detected in an additional four replicates at 1.80 log_10_ parasites/mL and nine replicates at 1.50 log_10_ parasites/mL. Linear regression was used to estimate the relationship between log_10_ concentration and C_q_ value with intercept of 43.370 (95% CI 43.097, 43.643), slope of − 3.353 (95% CI − 3.425, − 3.281), mean squared error of 0.088 and correlation coefficient (R^2^) of 0.997. There was no evidence of non-linear behaviour in the residual plot (Additional file 1: Figs. S1 and S2). The qPCR efficiency was 99%. The reliable reportable range of the 18S rRNA gene qPCR assay was 1.50 to 6.50 log_10_ parasites/mL.

### Precision

#### Intra-assay variability

Variability between replicates pooled across days increased as the standard concentration decreased, but the %CV remained low (i.e. < 3%) (Additional file 1: Table S3). The overall SD was 0.456 C_q_ units (0.137 log_10_ parasites/mL) pooled across all concentrations, and the overall %CV was less than 3% (1.45%). All negative controls were ND.

#### Inter-assay variability

The SD between studies increased as standard concentration decreased; however, variability was low (CV < 3%) and stable across concentrations (Additional file 1: Table S4). The overall SD between studies was 0.604 C_q_ units (0.182 log_10_ parasites/mL) pooled across all qPCR. The Overall SD between cohorts was 1.71 C_q_ units, reflecting that environmental and temporal variability was much higher than both intra-assay and inter-assay SD but remained stable across concentrations.

### Accuracy

**Fig. 1:**
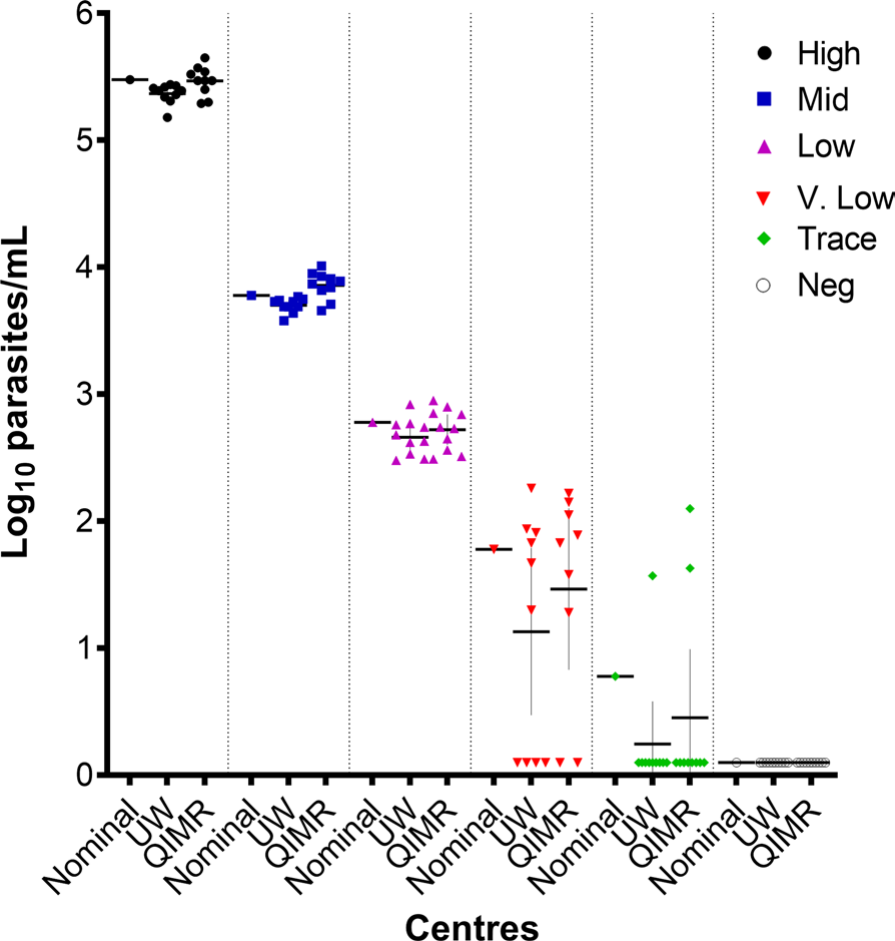
Comparison of nominal log_10_ parasite concentration to University of Washington and QIMR values. Nominal and University of Washington data adapted from Fig. 1 of [38]. Concentrations include high (5.487 log10 parasites/mL), mid (3.78 log10 parasites/mL), low (2.78 log10 parasites/mL), very low (1.78 log10 parasites/mL), trace (0.78 log10 parasites/mL) and neg (negative control)

The QIMR 18S rRNA gene qPCR assay detected 7/9 samples at the very low (1.78 log_10_ parasites/mL) level and 2/11 samples at the trace (0.78 log_10_ parasites/mL) level (Fig. 1). There were no false positives among the 10 blinded malaria-negative samples. The QIMR assay quantitatively aligned with both nominal values and with host laboratory RT-qPCR-generated parasitaemia (Additional file 1: Fig. S3). Quantitative bias for the QIMR 18S rRNA gene qPCR assay was minimal: + 0.019 log_10_ parasites/mL against the nominal values and + 0.104 log_10_ parasites/mL against the UW RT-qPCR.

The QIMR assay binds to three of the five 18S rRNA gene copies present in *P. falciparum* 3D7 strain located in chromosome 1, 11 and 13. Using the PlasmoDB platform, 20 SNPs scattered throughout these three chromosomes were identified. None of the SNPs were present in the binding regions or either the primers or probe.

### Specificity

BLAST searching (conducted on 7th April 2020) confirmed the 18S rRNA gene qPCR assay primer and probe sequences are specific to the *P. falciparum* 18S rRNA gene and do not cross-react with human genomic DNA or other *Plasmodium* species, including *P. ovale*, *P. malariae*, *P. vivax* and *P. knowlesi*. Likewise, the 18S rRNA gene qPCR assay provided negative results for all duplicates of blood samples from all 164 malaria-naïve VIS participants prior to inoculation. Thus, the analytical and diagnostic specificity were both determined to be 100%.

The effects of haemoglobin and EDTA as potential PCR inhibitory substances were examined. The differences between the means of parallel spiked blood and PBS extracts were compared. C_q_ values from both 18S and EHV qPCR obtained from two sample types were not significantly different (18S mean C_q_ difference = − 0.136, p = 0.14; EHV mean C_q_ difference = 0.134, p = 0.064). Therefore, there was no significant influence on the assay attributed to the presence of haemoglobin and EDTA.

## Discussion

Estimates of parasitaemia in malaria-naïve participants represent key data in VIS [15, 44]. This study reports the high sensitivity, reproducibility, and accuracy of a qPCR method to detect *P. falciparum* in blood samples. The clinical trial dataset used to assess parasitaemia included 786 assay runs across 30 clinical trials. This 18S rRNA gene qPCR assay was shown to have excellent analytic sensitivity and accuracy compared to the nominal value in the EQA study [38]. The ability to quantify low level parasitaemia allows for a greater increase in the safety of participants and for more accurate assessment of early recrudescence.

The LOD reported in this study is more precise than the LOD reported by Rockett et al. [28] (2.045 log_10_ parasites/mL versus 1.806 log_10_ parasites/mL). This is a consequence of the use of a larger number of replicates of the standard material over a wider range of dilutions [45] and the 95th percentile of the probit curve is used here whereas the 50th percentile of the lowest standard concentration was used in the earlier study. It is worth noting that LLoQ, the lowest standard concentration, was reported in this study instead of LoQ and that the LLoQ had a value lower than the LOD. VIS studies are unique in that the volunteers are tested and confirmed malaria negative upon enrolment and the positivity of blood samples is due to inoculation of the volunteer with malaria parasites. Any positive results, regardless of the quantification value, is of clinical significance and ensures volunteer safety in VIS studies. Although LOD is an important measure of diagnostic accuracy, it is less relevant in the context of VIS studies and the reportable range, specified by LLoQ and ULoQ, is most relevant for this application. C_q_ values that translate to positive findings lower than the LLoQ (1.5 log_10_ parasites/mL) have been observed in QIMR studies, particularly at the time when parasites first emerge in peripheral blood and during the “tail phase” after anti-malarial treatments. Detection and quantification below the defined reportable range remains plausible due to stochastic sampling, but high levels of variability should be expected due to the increase in stochastic effect on sampling and amplification at low target concentrations [22, 46]. By triplicate qPCR testing in clinical trials, this variation at low concentrations can be quantified. Approaches to further increase sensitivity include increasing sample volume input [47] or RT-qPCR measuring the more abundant 18S rRNA transcripts [48].

The 18S rRNA gene qPCR assay described here was designed and validated for the quantification of specific strains of *P. falciparum* (including NF54/3D7). The protocol and methodology were designed specifically for the purpose of this VIS setting. Using the current assay to quantify parasitaemia in field-collected samples may present separate challenges, such as ensuring sample integrity and the presence of circulating gametocytes. Ballard et al*.* [49] have previously shown the QIMR assay to give equivalent parasitaemia estimates to those from microscopy in field samples.

Assay validation is key to ensuring comparable accuracy among research centres. Individual standard protocols were used by each of the five participating centres in the EQA study [38] and QIMR results were comparable at higher concentrations and with higher sensitivity at lower concentrations. The QIMR assay presented consistently low intra-assay variability and inter-assay variability and was specific to the target, all of which are essential ingredients to VIS.

The main disadvantage of qPCR assays in a long-running clinical trial program is the need for a standard curve, because of variation between controls and the need for constant generation of new biological material. An alternative is droplet digital PCR (ddPCR). ddPCR allows direct quantification without the need for a standard curve. However, this approach is costly and not widely available [50].

Although multiple qPCR and RT-qPCR assays have been reported in the context of quantitating *P. falciparum* parasitaemia, the processes involved in the pre-analytical and analytical steps will affect the final outcome. Thus, because of its rigorous validation, this study represents a robust DNA-based method for quantifying malaria parasites with the overarching aim to contribute to a future standardized guideline for quantification of malaria parasites in a variety of settings.

## Conclusion

This study describes a comprehensive validation on a real-time qPCR assay using a hydrolysis probe for quantification of *P. falciparum* parasites in clinical trials. The assay was found to be highly sensitive and specific and able to produce reliable and reproducible results in agreement with international laboratories, thereby providing accurate estimation of parasite load for parasite growth and clearance rate before, during and after anti-malarial treatment. The assay is considered fit for purpose as an analytically validated qPCR assay to be used in malarial clinical trials.

## Supplementary Information

**Additional file 1: Table S1**. Oligonucleotide sequences used in this study. **Table S2.** List of VIS included in validation. **Table S3**. Summary of C_q_ values of each standard of 7 technical replicates over 3 consecutive days. **Table S4.** Summary of inter-assay variability for standards data for 18S rRNA gene qPCR assays. **Figure S1.** Scatter plot of C_q_ value for each log_10_ concentration of calibrator. **Figure S2.** Residual plot for the linear regression of log_10_ concentration of calibrator predicting C_q_. **Figure S3.** Bland–Altman plots comparing: (A) QIMR qPCR versus nominal, (B) QIMR qPCR versus UW RT-qPCR.

## Data Availability

The datasets analysed during the current study are available from the corresponding author on request.

## Abbreviations

%CV: Coefficient of variation
ddPCR: Droplet digital PCR
EDTA: Ethylenediaminetetraacetic acid
EHV: Equine herpesvirus
EQA: External quality assurance
LOD: Limit of detection
LoQ: Limit of quantification
MIQE: Minimum information for publication of quantitative real-time PCR experiments
PBS: Phosphate buffered saline
qPCR: Quantitative polymerase chain reaction
qRT-PCR: Quantitative real-time PCR
RBC: Red blood cell
rRNA: Ribosomal RNA
RT-qPCR: Reverse transcription qPCR
SD: Standard deviation
VIS: Volunteer infection studies
