# Supplementary material for: Analytical validation of a real-time hydrolysis probe PCR assay for quantifying Plasmodium falciparum parasites in experimentally infected human adults
Source: Malar J. 2021 Apr 10;20:181. doi: 10.1186/s12936-021-03717-y (PMC8035755; doi:10.1186/s12936-021-03717-y)
Supplement: Supplementary file 1 — Additional file 1: Table S1. Oligonucleotide sequences used in this study. Table S2. List of VIS included in validation. Table S3. Summary of Cq values of each standard of 7 technical replicates over 3 consecutive days. Table S4. Summary of inter-assay variability for standards data for 18S rRNA gene qPCR assays. Figure S1. Scatter plot of Cq value for each log10 concentration of calibrator. Figure S2. Residual plot for the linear regression of log10 concentration of calibrator predicting Cq. Figure S3. Bland–Altman plots comparing: (A) QIMR qPCR versus nominal, (B) QIMR qPCR versus UW RT-qPCR. [file 12936_2021_3717_MOESM1_ESM.docx]

**Supplementary material**

**Table S1**. Oligonucleotide sequences used in this study.

| *P. falciparum* 18S rRNA gene (1) | |
| --- | --- |
| PerFal forward | 5’- CTTTTGAGAGGTTTTGTTACTTTGAGTAA -3’ |
| PerFal reverse | 5’- TATTCCATGCTGTAGTATTCAAACACA -3’ |
| PerFal probe | 5’- FAM-TGTTCATAACAGACGGGTAGTCATGATTGAGTTCA-BHQ1-3’ |
| Equine herpesvirus (EHV) (2) | |
| EHV forward | 5’- GATGACACTAGCGACTTCGA -3’ |
| EHV reverse | 5’- CAGGGCAGAAACCATAGACA -3’ |
| EHV probe | 5’- QUASAR670 -TTTCGCGTGCCTCCTCCAG-BHQ1-3’ |

**Table S2.** List of VIS included in validation.

| **Number** | **Study cohort** |
| --- | --- |
| 1 | ACTRN12613000533796 Cohort 1 |
| 2 | ACTRN12613000533796 Cohort 2 |
| 3 | ACTRN12613000565741 Cohort 1 |
| 4 | ACTRN12613000565741 Cohort 2 |
| 5 | ACTRN12613000565741 Cohort 3A |
| 6 | ACTRN12613000565741 Cohort 3B |
| 7 | ACTRN12613001040752 Cohort 1 |
| 8 | NCT02223871 Cohort 1 |
| 9 | NCT02389348 Cohort 1 |
| 10 | NCT02389348 Cohort 2 |
| 11 | NCT02431637Cohort 1 |
| 12 | NCT02543086Cohort 1 |
| 13 | NCT02431650 Cohort 1 |
| 14 | NCT02573857 Cohort 1 |
| 15 | NCT02431637 Cohort 3 |
| 16 | NCT02431650 Cohort 2 |
| 17 | NCT02431650 Cohort 2B |
| 18 | NCT02431650 Cohort 3 |
| 19 | NCT02867059 Cohort 1 |
| 20 | NCT02867059 Cohort 2 |
| 21 | NCT02867059 Cohort 2B |
| 22 | NCT02783833 Cohort 1 |
| 23 | NCT02783833 Cohort 2 |
| 24 | NCT03261401 Cohort 1 |
| 25 | NCT03261401 Cohort 2 |
| 26 | ACTRN12617001394336 Cohort 1 |
| 27 | ACTRN12617001394336 Cohort 2 |
| 28 | ACTRN12617001394336 Cohort 3 |
| 29 | NCT03542149 Cohort 1 |
| 30 | NCT03542149Cohort 2 |

**Table** **S3**. Summary of C_q_ values of each standard of 7 technical replicates over three consecutive days.

| **Concentrations (log_10_ parasites/mL)** | **Positive/total replicates tested (%)** | **Mean C_q_ (SD)** | **C_q_ range (min, max)** | **C_q_ %CV** |
| --- | --- | --- | --- | --- |
| 6.50 | 21/21 (100) | 21.38 (0.141) | 21.11 - 21.77 | 0.66 |
| 5.50 | 22/22 (100) | 25.03 (0.235) | 24.49 - 25.81 | 0.94 |
| 4.50 | 21/21 (100) | 28.29 (0.138) | 28.06 - 28.60 | 0.49 |
| 3.50 | 21/21 (100) | 31.57 (0.227) | 31.05 - 31.98 | 0.72 |
| 2.80 | 21/21 (100) | 33.93 (0.361) | 33.28 - 34.51 | 1.06 |
| 2.50 | 21/21 (100) | 35.34 (0.523) | 34.35 - 36.34 | 1.48 |
| 2.20 | 20/21 (95) | 36.23 (0.480) | 35.27 - 36.95 | 1.32 |
| 1.80 | 16/21 (76) | 37.22 (0.836) | 35.89 - 38.67 | 2.25 |
| 1.50 | 12/21 (57) | 37.90 (0.667) | 36.90 - 39.09 | 1.76 |
| Negative control* | 0/21 (0) | Not detected | n/a | n/a |
| Total | 175 | 31.38 (0.456^) | 21.11, 39.09 | 1.45 |

^Overall Standard deviation (SD); *Negative control: uninfected human blood extracts; n/a = not applicable

**Table S4.** Summary of inter-assay variability for standards data for 18S rRNA gene qPCR assays.

| **Concentrations (log_10_ parasites/mL)** | **Positive/total replicates tested (%)** | **Mean C_q_ (SD)** | **SD (Cohort)** | **%CV** |
| --- | --- | --- | --- | --- |
| 6.50 | 786/786 (100) | 20.63 (0.397) | 1.60 | 1.92 |
| 5.50 | 786/786 (100) | 24.04 (0.440) | 1.61 | 1.83 |
| 4.50 | 786/786 (100) | 27.52 (0.447) | 1.64 | 1.62 |
| 3.50 | 785/785 (100) | 31.07 (0.502) | 1.69 | 1.62 |
| 2.50 | 781/786 (99) | 34.77 (0.866) | 2.05 | 2.49 |
| 1.50 | 379/786 (48) | 37.11 (0.980) | 1.60 | 2.64 |
| Negative control | 0/786 (0) | Not-detected | n/a | n/a |
| Overall SD |  | 0.604 | 1.71 |  |

SD = standard deviation; %CV = coefficient of variation; n/a = not applicable

**Figure S1:** Scatter plot of C_q_ value for each log_10_ concentration of calibrator.

**Figure S2:** Residual plot for the linear regression of log_10_ concentration of calibrator predicting C_q_ value.


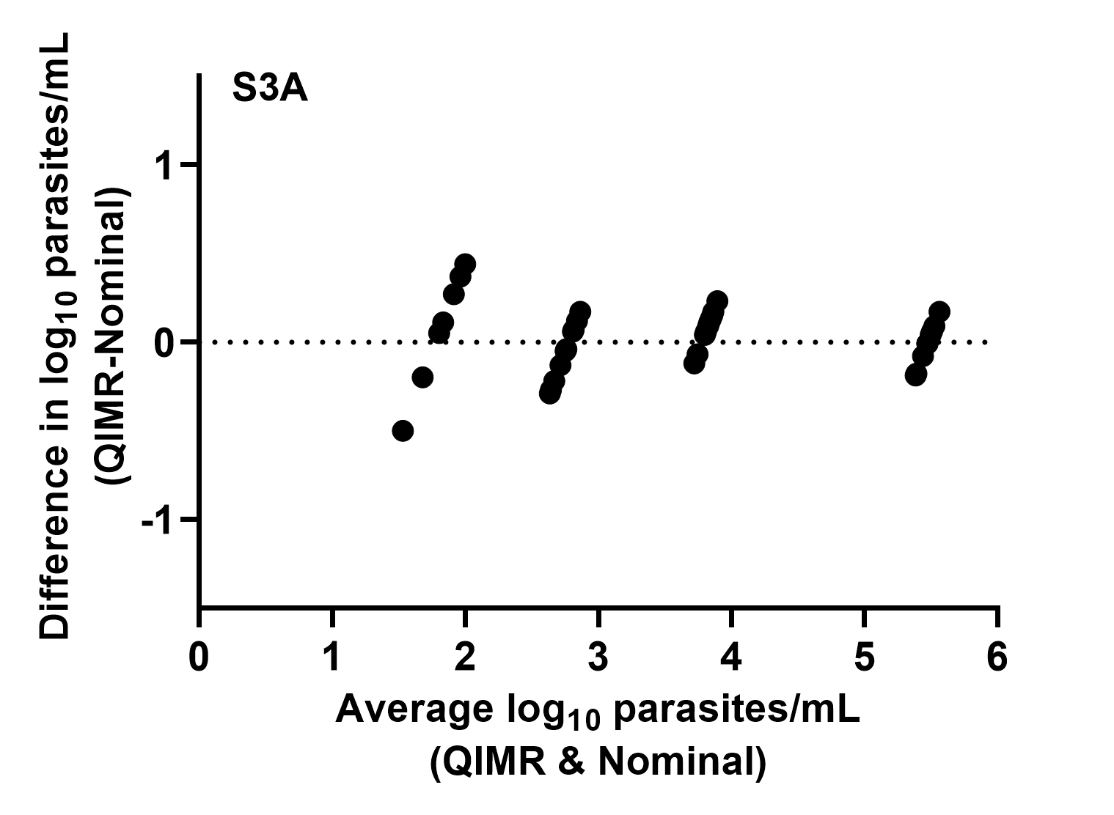


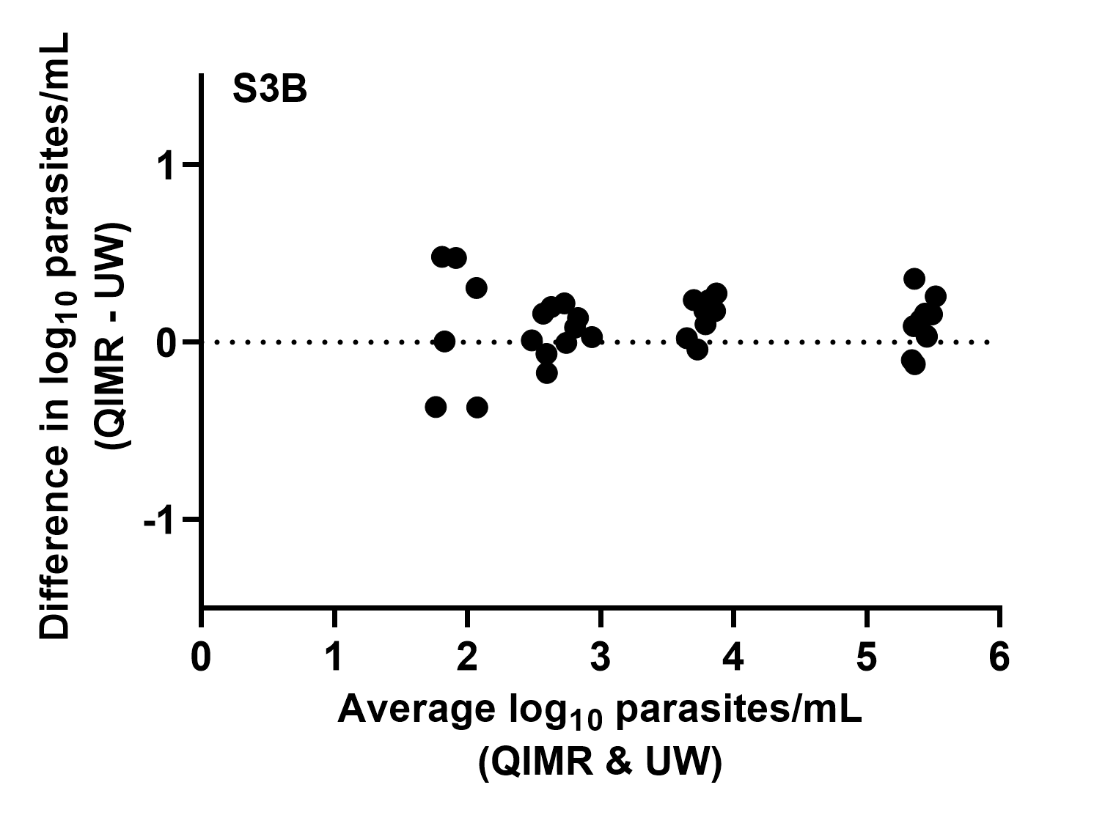


**Figure S3:** Bland-Altman plots comparing: A) QIMR qPCR versus nominal, B) QIMR qPCR versus UW RT-qPCR.

**References**

1. Rockett RJ, Tozer SJ, Peatey C, Bialasiewicz S, Whiley DM, Nissen MD, Trenholme K, Mc Carthy JS, Sloots TP: A real-time, quantitative PCR method using hydrolysis probes for the monitoring of *Plasmodium falciparum* load in experimentally infected human volunteers. Malar J. 2011;10:48.
2. Bialasiewicz S, Whiley DM, Buhrer-Skinner M, Bautista C, Barker K, Aitken S, Gordon R, Muller R, Lambert SB, Debattista J, et al: A novel gel-based method for self-collection and ambient temperature postal transport of urine for PCR detection of chlamydia trachomatis. Sex Transm Infect. 2009;85:102-105.
